# Supplementary material for: Jaxley: differentiable simulation enables large-scale training of detailed biophysical models of neural dynamics
Source: Nat Methods. 2025 Nov 13;22(12):2649–57. doi: 10.1038/s41592-025-02895-w (PMC12695658; doi:10.1038/s41592-025-02895-w)
Supplement: Supplementary file 1 — Supplementary Discussion, Results, Table 1 and Figs. 1–3. [file 41592_2025_2895_MOESM1_ESM.pdf]

nature methods

Supplementary information

https://doi.org/10.1038/s41592-025-02895-w

JAXLEY: differentiable simulation enables large-scale training of detailed biophysical models of neural dynamics

In the format provided by the authors and unedited

Contents

Comparison to other toolboxes . . . 2
Details on the numerical solver . . . 2
Training overview . . . 2
Details on parameter bounds and initialization . . . 3
Dynamic time warping . . . 4
Hamiltonian Monte–Carlo . . . 4
Data preprocessing for the retina model . . . 4
Computing Lyapunov exponents . . . 5
Supplementary figures . . . 6

Comparison to other toolboxes

Over the past decades, neuroscientists have built a wide range of neuroscience simulators. Several simulators enable scientists to construct biophysically and morphologically detailed models (e.g., NEURON [1], NetPyNE [2], or Arbor [3]). However, these toolboxes are typically written in programming languages that do not support automatic differentiation and backpropagation of error. With the development of machine learning toolboxes such as PyTorch, Keras or JAX, scientists have built neuroscience simulators on top of these toolboxes (e.g., BindsNET [4], SNNTorch [5], Norse [6], or BrainPy [7]). These toolboxes simulate simplified spiking neural networks (SNNs, typically with leaky-integrate-and-fire dynamics) or point neuron models and enable training those models with (surrogate) gradient descent. However, these toolboxes cannot incorporate biophysical and morphological detail. JAXLEY is the only toolbox which enables simulation of biophysically detailed neuron models and permits backpropagation of error. JAXLEY can simulate cells and networks with morphological and biophysical detail, including models with diverse membrane and synaptic mechanisms and intracellular dynamics such as ion diffusion. Backpropagation of error enables training the biophysical parameters describing these simulations on large datasets and it overcomes previous limits on the number of parameters.

Details on the numerical solver

To solve the differential equations, we performed a leapfrog update of voltage equations and gate equations. As is also done in NEURON, at every time step, the current through mechanisms (channels and synapses) was evaluated twice at voltage values that differ by 0.001 mV. This allowed to infer the voltage-dependent and voltage-independent contributions to the current dynamics (which is required by the implicit Euler solver of the voltage equations). In order to achieve a high degree of parallelization, we modelled every branch in the cell (or network) with four compartments (with the exception of the network shown in Fig. 1f, for which we used two compartments). We split branches that were longer than 300 µm into sub-branches until each sub-branch was shorter than 300 µm. We note that JAXLEY also supports different methods for discretization (e.g., the d-lambda rule) and different solvers for the tridiagonal system.

Training overview

In Table 1, we list the training procedure for all tasks, including the number of optimized parameters, the number of branches that the model has (note that each branch is modelled with 4 compartments), the compute device we trained on, the number of gradient steps we took to arrive at the model shown in the figures, and the compute time it took to perform one gradient step.

Table 1. Overview of tasks and their training procedure.

Task	params	branches	device	gradient steps	time per step
L5PC synthetic (Fig. 2b)	19	339	CPU	10	5 sec
Allen cell 1 (Fig. 2d, left)	25	103	GPU, H100	50	150 sec
Allen cell 2 (Fig. 2d, right)	25	99	GPU, H100	50	150 sec
Fit conductance profiles (Fig. 2f)	1390	339	CPU	500	0.04 sec
Nonlinear neuron (Fig. 2g)	72	3	CPU	6400	0.0017 sec
Hybrid retina model (Fig. 3)	607	147	GPU, A100	560	55 sec
RNN for evidence integration (Fig. 4)	109	62	CPU	2000	12 sec
Delayed-match-to-sample task (Fig. 4)	459	152	GPU	1000	36 sec
Biophysical network for MNIST (Fig. 5)	105k	2000	GPU, V100	26k	28 sec

Details on parameter bounds and initialization

Fitting single cell models to voltage recordings

We used the same parameter search bounds as Van Geit et al. [8] for the synthetic data, but we enforced that somatic potassium existed (lower bound 0.25 mS/cm^2). For the experimental recordings from the Allen Cell Types Database, we used slightly larger bounds for three of the parameters to increase the flexibility of the model: We used bounds of [0, 6] mS/cm^2 (instead of [0, 4] mS/cm^2) for somatic and axonal sodium channels and a lower bound of 1 ms for the delay of the calcium buffer (instead of 20 ms). In addition, we made the following six parameters trainable: To account for the fact that channel models were built upon measurements from other cells, we allowed a fivefold variation of the time constant of the high- and low-voltage-activated calcium channels and the M channel, and we introduced a parameter that can shift the activation curve of the somatic sodium channel by up to 10 mV to the left. In addition, to account for ion channels typically being selective to more than a single ion, we allowed the potassium reversal potential to vary within [-100, -70] mV and the sodium reversal potential to vary within [40, 60] mV. To fit recordings from the Allen Cell Types Database (IDs 485574832, 488683425, 480353286, and 473601979), we modified the leak conductance to 10^{-4} for all cells, the leak reversal potential to -88, -88, -95, and -95 mV, and capacitance to 6, 2, 6, and 2.5 µF/cm^2, respectively.

Fitting voltage recordings of all branches

Parameters had the same lower and upper bound as for the L5PC described above.

Nonlinear single neuron computation

Parameters were initialized randomly within uniform bounds. The bounds were [0.05, 1.1] for sodium, [0.01, 0.3] for potassium, and [0.0001, 0.001] for leak, all in mS/cm^2. The bounds for the radius were [0.1, 5.0] µm, for the length [1, 20] µm per compartment, and for the axial resistivity [500, 5500] Ωcm.

Hybrid model of the retina

We initialized all membrane conductances at previously published values [9], with an exception of sodium conductances which we initialized at 0.15 in the soma and 0.05 in the dendrite. We sampled the initial synaptic strengths randomly within 0 and 0.1 nS, and then divided the synaptic conductance by the number of postsynaptic connections that a bipolar cells makes (such that, in expectation, every BC has the same impact on the RGC). We initialized the axial resistivity of every compartment at 5,000 Ωcm. Finally, we initialized the radius of every dendritic compartment at 0.2 µm. We kept the diameter of the soma constant at 10 µm.

We used the following bounds for optimization: For somatic conductances we used [0.05, 0.5] for sodium, [0.01, 0.1] for potassium, [10^{-5}, 10^{-3}] for leak, [0.01, 0.1] for transient potassium, [$2{\cdot}10^{-5}$, $2{\cdot}10^{-4}$] for calcium dependent potassium, and [0.002, 0.003] for calcium. All membrane conductance units are mS/cm^2. For dendritic conductances, we used the same bounds apart from the lower bound of 0 for sodium. For the branch radii, we used bounds of [0.1, 1.0] µm [10]. For the axial resistivities, we used [100, 10,000] Ωcm. For the synaptic conductances, we used [0.0, 0.2] nS.

A recurrent neural network of biophysical neurons performing an evidence integration task

Initial values for the maximum synaptic conductances were drawn from a standard normal distribution scaled by an initial gain g such that the bulk of the eigenspectrum of the synaptic weights lay in a circle on the complex plane with radius g (after multiplying inhibitory synapse weights by -1). We presented inputs by stimulating neurons at their basal dendrite. We set $g = 5.0\pi \;/\; 5 \cdot 10^3$, which is close to the transition point between stable and chaotic dynamics. For recurrent connections, we set the rate constant for transmitter-receptor dissociation rate (the $k\_$ parameter [11]), that influences the synaptic time-constants, to 1/1.0 ms. For connections to the rate-based readout neurons, we used slower synapses, with $k\_$ set to 1/40 ms. All recurrent units received stimulus input scaled by random initial weights drawn from $\mathcal{N}(0, 0.1)$. We used bounds of [-0.2, 0.2] for the input weights and [0, $3 \cdot \mathrm{max}(g)$] for the maximal synaptic conductances.

A recurrent neural network of biophysical neurons performing a delayed-match-to-sample task

We trained the maximal synaptic conductances, constrained to the range [0, ∞), using a SoftPlus function. We also trained the weights of the stimulus input to each neuron in the network, restricted to the range [0, 4], as well as $k\_$ restricted to [0.05, 2].

We set $g = 5.0\pi \ / \ 5 \cdot 10^3$. We set the $k\_$ parameter of recurrently connected neurons to 1, and to 0.1 for the slower synapses onto the readout neurons. The connection probability from input to (the basal dendrite of) recurrent units was 0.1, with initial weights drawn from $\mathcal{U}_{[0,1]}$.

A biophysical network that performs computer vision tasks

We used the following bounds for the parameters: [0.05, 0.5] for sodium, [0.01, 0.1] for potassium, [0.0001, 0.001] for leak, [-5 / 28^2 / 25, 5 / 28^2 / 25] for synapses from the input to the hidden layer and [-5 / 64 / 25 / 2, 5 / 64 / 25 / 2] for synapses from the hidden layer to the output layer. We initialized sodium maximal conductances at 0.12 mS/cm^2, potassium at 0.036 mS/cm^2, and leak at 0.0003 mS/cm^2. We initialized synaptic conductances as samples from a Gaussian distribution with mean 0 and standard deviation 1 / 28^2 / 25 for the first layer and standard deviation 1 / 64 / 25 / 2 for the second layer. We set the synaptic rate constant for transmitter-receptor dissociation to $k_- = 1/4$.

Dynamic time warping

To implement soft dynamic time warping (soft-DTW), we preprocess the raw voltage traces by applying a sliding window max reduction with a window size of 50 timesteps and a stride of 30 timesteps (each timestep is 0.025 ms). This step reduces the number of time points while preserving the geometric shape of action potentials. We then rescale both the observations and simulations to the unit interval using the same scaling factors. We use the cost function $c(x_i, y_i) = |x_i - y_i| + |i - j|$, where i and j are time points and x and y are voltage traces. The first term penalizes deviations in the voltage trace while the second term introduces a shift penalty that encourages pointwise alignment. We apply this loss function to the reduced traces to compute the discrepancy between simulated and experimentally observed voltage traces.

Hamiltonian Monte–Carlo

We used Hamiltonian Monte–Carlo (HMC) to perform Bayesian inference across conductance profiles of single neurons. Since the optimization is performed in unconstrained space, we also conducted HMC in this unconstrained space through a change of variables. We utilized a Laplace log-likelihood with a scale parameter of $\lambda = 0.001$, ensuring that the unnormalized posterior log density matched the loss function of the optimization problem. We used the BlackJAX [12] implementation of HMC. For each step, we performed five integration steps for the Hamiltonian dynamics with a step size of 0.01, leading to an average acceptance rate of $\approx 65\%$.

To visualize the learned conductance profile, we discretized distance from soma into eleven bins and grouped all parameters within each bin. We then calculated histograms for each of the bins and generated a spline interpolation of all quantile lines (Fig. 2h).

Data preprocessing for the retina model

To denoise the calcium data, we lowpass-filtered the raw calcium data with a butterworth filter and a cutoff frequency of 7 Hz. We z-scored the resulting signal, with a different mean and standard deviation for each region of interest.

Next, we generated a single label for each image. We did this by performing a linear regression from image onto delayed calcium signals, and then used the calcium at the delay which was most predictive (i.e., had highest Pearson correlation between linear regression prediction and data). This led us to a delay of 1.8 seconds. As label, we used the low-pass filtered calcium value after this delay (starting from image onset).

We followed Ran et al. [10] to compute receptive fields. We used automatic smoothness detection (ASD) [13] with 20 iterations of evidence optimization. We standardized all receptive fields to range from 0 to 1 and, for contours, thresholded the receptive fields at a value of 0.6.

Computing Lyapunov exponents

We can quantify the stability of recurrent networks by measuring the average rate of divergence or convergence of nearby trajectories, which is given by the maximal Lyapunov exponent. To obtain the maximal Lyapunov exponent, we first discretised our model to obtain $\mathbf{x}_{t+1} = \mathbf{f}(\mathbf{x}_t)$, where $\mathbf{x}_t$ is a vector of all dynamic variables (e.g., voltages, gating variables) at time t, and $\mathbf{f}$ is one step of the chosen solver. We can then define the maximal Lyaponuv exponent as: $\lambda_{\max}(\mathbf{x}_0) = \lim_{t\to\infty} \frac{1}{t} \lim_{\epsilon\to 0} \log \frac{\|\epsilon \mathbf{u}_t\|}{\|\epsilon \mathbf{u}_0\|}$ where $\mathbf{u}_0$ is a perturbation to the initial state of the system $\mathbf{x}_0$. We measured the evolution of infinitesimal perturbations to $\mathbf{x}_{1:T}$ by calculating the Jacobian at each point along a trajectory.

We used the following numerical algorithm to approximate $\lambda_{\max}(\mathbf{x}_0)$ [14]: First, we generated an initial state $\mathbf{x}_0$ and initial unit norm vector $\mathbf{q}_0$. After discarding initial transients for 4 seconds of simulation, we let the system $\mathbf{x}_{t+1} = \mathbf{f}(\mathbf{x}_t)$ and $\mathbf{q}_{t+1} = D\mathbf{f}|_{\mathbf{x}_t}\mathbf{q}_t$ (where D denotes the Jacobian) evolve for $T = 2.4 \cdot 10^5$ timestep (a further 6 seconds). Note that $\mathbf{q}_{t+1}$ can be efficiently computed using Jacobian vector products in JAX [15]. At every timestep we computed $r_t = \|\mathbf{q}_t\|$ and renormalised: $\mathbf{q}_t \leftarrow \frac{\mathbf{q}_t}{r_t}$. The maximal Lyaponuv exponent was then given by $\frac{1}{T}\sum_1^T \log \|r_t\|$.

Supplementary figures

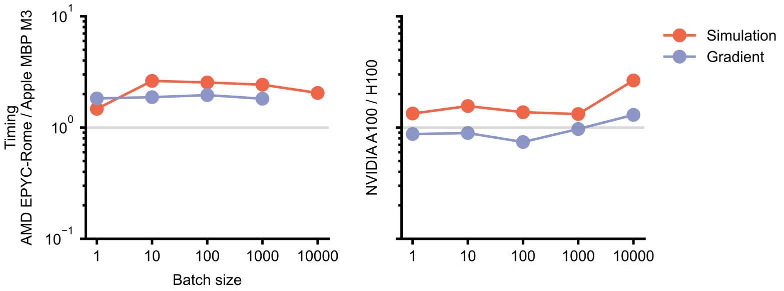

Figure S1. Runtimes on two additional compute platforms for the morphology and setup shown in Fig. 1e. Left: Runtime on AMD EPYC-Rome (shown in main text) divided by runtime on Apple Macbook Pro M3. JAXLEY runs about two times faster on a Macbook Pro as compared to the AMD CPU. The missing datapoint for the gradient at batch size 10k is due to the limited memory of the Macbook Pro. Right: Same as left, but dividing the runtime on an NVIDIA A100 (shown in the main text) by the runtime on an NVIDIA H100. The H100 is faster for simulation and gradient calculation for large batch sizes. The A100 was slightly faster for computing the gradient for small batch sizes, but we expect that this difference stems from differences in the storage system between the A100 and H100 on our compute cluster.

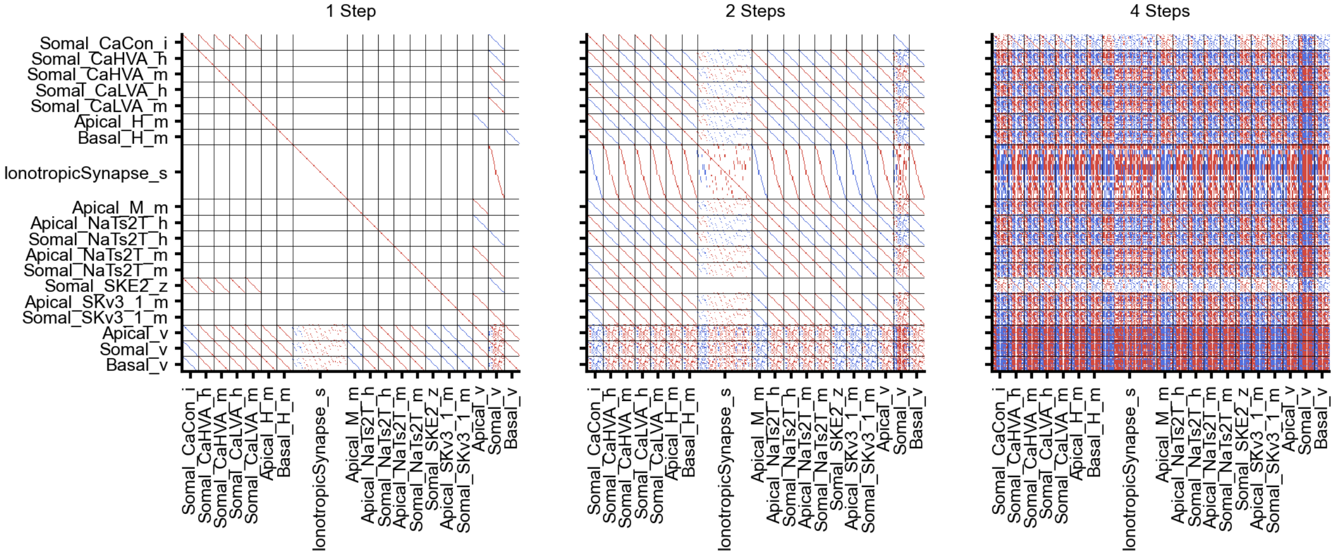

Figure S2. Jacobians reveal interactions between states JAXLEY allows us to compute Jacobians of biophysical networks by using automatic differentiation. Here we show $D\mathbf{f}|_{\mathbf{x}_0}$, where $D\mathbf{f}$ denotes the Jacobian with respect to $\mathbf{f}$, and $\mathbf{f}$ is 1, 2, and 4 steps of simulation with initial state $\mathbf{x}_0$. We used a recurrent network similar to those used in Fig. 4 (here: 20 units of which 5 are inhibitory; connection probability 0.2). As the scale between different states can be very different, we here just show the sign (red is positive, white is zero, and blue is negative).

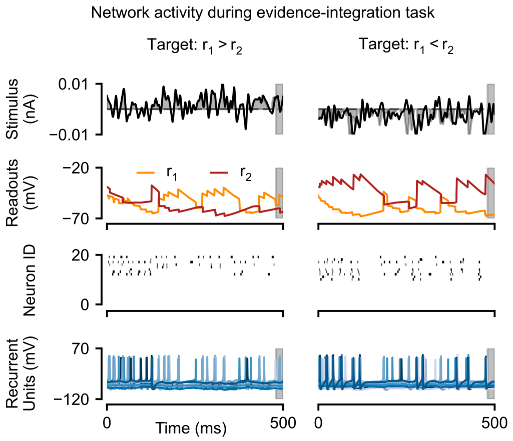

Figure S3. Spiking activity during evidence integration task. Task input current (first row), readout activity (second row), spike raster (third row), and voltage activity of recurrent neurons (fourth row) during the evidence integration task, after training.

References

[1] Nicholas T Carnevale and Michael L Hines. *The NEURON book*. Cambridge University Press, 2006.

[2] Salvador Dura-Bernal, Benjamin A Suter, Padraig Gleeson, Matteo Cantarelli, Adrian Quintana, Facundo Rodriguez, David J Kedziora, George L Chadderdon, Cliff C Kerr, Samuel A Neymotin, et al. NetPyNE, a tool for data-driven multiscale modeling of brain circuits. *Elife*, 8:e44494, 2019.

[3] Nora Abi Akar, Ben Cumming, Vasileios Karakasis, Anne Küsters, Wouter Klijn, Alexander Peyser, and Stuart Yates. Arbor—a morphologically-detailed neural network simulation library for contemporary high-performance computing architectures. In *2019 27th euromicro international conference on parallel, distributed and network-based processing (PDP)*, pages 274–282. IEEE, 2019.

[4] Hananel Hazan, Daniel J Saunders, Hassaan Khan, Devdhar Patel, Darpan T Sanghavi, Hava T Siegelmann, and Robert Kozma. BindsNET: A machine learning-oriented spiking neural networks library in Python. *Frontiers in neuroinformatics*, 12:89, 2018.

[5] Jason K Eshraghian, Max Ward, Emre Neftci, Xinxin Wang, Gregor Lenz, Girish Dwivedi, Mohammed Bennamoun, Doo Seok Jeong, and Wei D Lu. Training spiking neural networks using lessons from deep learning. *Proceedings of the IEEE*, 111(9): 1016–1054, 2023.

[6] Christian Pehle and Jens Egholm Pedersen. Norse - A deep learning library for spiking neural networks, January 2021.

[7] Chaoming Wang, Tianqiu Zhang, Xiaoyu Chen, Sichao He, Shangyang Li, and Si Wu. BrainPy, a flexible, integrative, efficient, and extensible framework for general-purpose brain dynamics programming. *Elife*, 12, 2023.

[8] Werner Van Geit, Michael Gevaert, Giuseppe Chindemi, Christian Rössert, Jean-Denis Courcol, Eilif B Muller, Felix Schürmann, Idan Segev, and Henry Markram. BluePyOpt: leveraging open source software and cloud infrastructure to optimise model parameters in neuroscience. *Frontiers in neuroinformatics*, 10:17, 2016.

[9] JF Fohlmeister and RF Miller. Impulse encoding mechanisms of ganglion cells in the tiger salamander retina. *Journal of neurophysiology*, 78(4):1935–1947, 1997.

[10] Yanli Ran, Ziwei Huang, Tom Baden, Timm Schubert, Harald Baayen, Philipp Berens, Katrin Franke, and Thomas Euler. Type-specific dendritic integration in mouse retinal ganglion cells. *Nature Communications*, 11(1):2101, 2020.

[11] Astrid A Prinz, Dirk Bucher, and Eve Marder. Similar network activity from disparate circuit parameters. *Nature neuroscience*, 7 (12):1345–1352, 2004.

[12] Alberto Cabezas, Adrien Corenflos, Junpeng Lao, and Rémi Louf. BlackJAX: Composable Bayesian inference in JAX, 2024.

[13] Maneesh Sahani and Jennifer Linden. Evidence optimization techniques for estimating stimulus-response functions. *Advances in neural information processing systems*, 15, 2002.

[14] Giancarlo Benettin, L. Galgani, Antonio Giorgilli, and Jean-Marie Strelcyn. Lyapunov characteristic exponents for smooth dynamical systems and for hamiltonian systems - a method for computing all of them. I - Theory. II - Numerical application. *Meccanica*, 15:21–30, 03 1980.

[15] James Bradbury, Roy Frostig, Peter Hawkins, Matthew James Johnson, Chris Leary, Dougal Maclaurin, George Necula, Adam Paszke, Jake VanderPlas, Skye Wanderman-Milne, and Qiao Zhang. JAX: composable transformations of Python+NumPy programs, 2018.
