# Supplementary material for: Jaxley: differentiable simulation enables large-scale training of detailed biophysical models of neural dynamics
Source: Nat Methods. 2025 Nov 13;22(12):2649–57. doi: 10.1038/s41592-025-02895-w (PMC12695658; doi:10.1038/s41592-025-02895-w)
Supplement: Supplementary file 2 — Reporting Summary [file 41592_2025_2895_MOESM2_ESM.pdf]

nature portfolio

Corresponding author(s): Michael Deistler, Jakob H. Macke

Last updated by author(s): Jun 12, 2025

Reporting Summary

Nature Portfolio wishes to improve the reproducibility of the work that we publish. This form provides structure for consistency and transparency in reporting. For further information on Nature Portfolio policies, see our Editorial Policies and the Editorial Policy Checklist.

Statistics

For all statistical analyses, confirm that the following items are present in the figure legend, table legend, main text, or Methods section.

n/a	Confirmed	
☐	☒	The exact sample size (*n*) for each experimental group/condition, given as a discrete number and unit of measurement
☒	☐	A statement on whether measurements were taken from distinct samples or whether the same sample was measured repeatedly
☐	☒	The statistical test(s) used AND whether they are one- or two-sided<br>*Only common tests should be described solely by name; describe more complex techniques in the Methods section.*
☒	☐	A description of all covariates tested
☐	☒	A description of any assumptions or corrections, such as tests of normality and adjustment for multiple comparisons
☐	☒	A full description of the statistical parameters including central tendency (e.g. means) or other basic estimates (e.g. regression coefficient) AND variation (e.g. standard deviation) or associated estimates of uncertainty (e.g. confidence intervals)
☐	☒	For null hypothesis testing, the test statistic (e.g. *F*, *t*, *r*) with confidence intervals, effect sizes, degrees of freedom and *P* value noted<br>*Give P values as exact values whenever suitable.*
☐	☒	For Bayesian analysis, information on the choice of priors and Markov chain Monte Carlo settings
☒	☐	For hierarchical and complex designs, identification of the appropriate level for tests and full reporting of outcomes
☒	☐	Estimates of effect sizes (e.g. Cohen's *d*, Pearson's *r*), indicating how they were calculated

Our web collection on statistics for biologists contains articles on many of the points above.

Software and code

Policy information about availability of computer code

Data collection	We used AllenSDK (v2.16.2) to obtain data from the Allen Cell Types Database (IDs 485574832, 488683425, 480353286, 473601979).<br><br>We downloaded CA1 neurons (Fig. 1d,f, Fig. 5) from Neuromorpho.org (the cell shown in Fig. 1d has ID NMO_00120, other cells are from the same archive and of the same cell type).<br><br>We downloaded the L5PC (Fig. 2) from BluePyOpt (v1.14.11, https://github.com/BlueBrain/BluePyOpt/blob/master/examples/l5pc/morphology/C060114A7.asc). We converted this file to SWC with the morph-tool software (https://github.com/BlueBrain/morph-tool).
Data analysis	We developed JAXLEY to perform biophysical simulations: https://github.com/jaxleyverse/jaxley<br>For the results shown in the paper, we used JAXLEY version 0.1.2.<br><br>Channel models are implementeed in our Jaxley-Mech library: https://github.com/jaxleyverse/jaxley-mech<br>For the results shown in the paper, we used version 0.1.0.<br><br>Experiments shown in the paper can be reproduced with the code provided here: https://github.com/mackelab/jaxley_experiments<br><br>We used JAX (v0.4.29), NumPy (v1.26.4), pandas (v2.2.1), and BlackJAX (v1.1.0) for data analysis and simulation.

For manuscripts utilizing custom algorithms or software that are central to the research but not yet described in published literature, software must be made available to editors and reviewers. We strongly encourage code deposition in a community repository (e.g. GitHub). See the Nature Portfolio guidelines for submitting code & software for further information.

Data

Policy information about availability of data

All manuscripts must include a data availability statement. This statement should provide the following information, where applicable:

- Accession codes, unique identifiers, or web links for publicly available datasets
- A description of any restrictions on data availability
- For clinical datasets or third party data, please ensure that the statement adheres to our policy

We used only publicly available data from the Allen Cell Types Database (https://celltypes.brain-map.org) and from Ran et al. (2020, available on Zenodo: https://zenodo.org/records/3708064). We used morphologies that are publicly available on NeuroMorpho (https://neuromorpho.org).

Research involving human participants, their data, or biological material

Policy information about studies with human participants or human data. See also policy information about sex, gender (identity/presentation), and sexual orientation and race, ethnicity and racism.

Reporting on sex and gender	Not applicable.
Reporting on race, ethnicity, or other socially relevant groupings	Not applicable.
Population characteristics	Not applicable.
Recruitment	Not applicable.
Ethics oversight	Not applicable.

Note that full information on the approval of the study protocol must also be provided in the manuscript.

Field-specific reporting

Please select the one below that is the best fit for your research. If you are not sure, read the appropriate sections before making your selection.

☒ Life sciences ☐ Behavioural & social sciences ☐ Ecological, evolutionary & environmental sciences

For a reference copy of the document with all sections, see nature.com/documents/nr-reporting-summary-flat.pdf

Life sciences study design

All studies must disclose on these points even when the disclosure is negative.

Sample size	For panel 3f, a total of seven independent datasets were used to compare the two architectures. No statistical methods were used to pre-determine this sample size. No other results performed statistical tests.
Data exclusions	No data was excluded.
Replication	For panel 3f, model training and evaluation were independently replicated across the seven datasets. Each dataset served as an independent test of model performance. A single statistical comparison (one-sided t-test) was performed across these seven runs.
Randomization	For panel 3f, datasets were selected independently of model performance and not influenced by experimental outcome.
Blinding	Blinding was not applicable to panel 3f, as model training and evaluation were fully automated.

Reporting for specific materials, systems and methods

We require information from authors about some types of materials, experimental systems and methods used in many studies. Here, indicate whether each material, system or method listed is relevant to your study. If you are not sure if a list item applies to your research, read the appropriate section before selecting a response.

Materials & experimental systems

n/a	Involved in the study
☒	☐ Antibodies
☒	☐ Eukaryotic cell lines
☒	☐ Palaeontology and archaeology
☒	☐ Animals and other organisms
☒	☐ Clinical data
☒	☐ Dual use research of concern
☒	☐ Plants

Methods

n/a	Involved in the study
☒	☐ ChIP-seq
☒	☐ Flow cytometry
☒	☐ MRI-based neuroimaging

Plants

Seed stocks	Not applicable.
Novel plant genotypes	Not applicable.
Authentication	Not applicable.
